# Upregulated WDR26 serves as a scaffold to coordinate PI3K/AKT pathway-driven breast cancer cell growth, migration, and invasion

**DOI:** 10.18632/oncotarget.7439

**Published:** 2016-02-17

**Authors:** Yuanchao Ye, Xiaoyun Tang, Zhizeng Sun, Songhai Chen

**Affiliations:** ^1^ Department of Pharmacology, Roy J. and Lucille A. Carver College of Medicine, University of Iowa, Iowa City, IA, USA; ^2^ Department of Internal Medicine, Roy J. and Lucille A. Carver College of Medicine, University of Iowa, Iowa City, IA, USA; ^3^ Current address: Department of Biochemistry, University of Alberta, Edmonton, AB, Canada; ^4^ Current address: Department of Pharmacology, Baylor College of Medicine, Houston, TX, USA

**Keywords:** WDR26, G protein-coupled receptors, PI3K, AKT, breast cancer growth and metastasis

## Abstract

The phosphatidylinositol 3-kinase (PI3K)/AKT pathway transmits signals downstream of receptor tyrosine kinases and G protein-coupled receptors (GPCRs), and is one of the most dysregulated pathways in breast cancer. PI3Ks and AKTs consist of multiple isoforms that play distinct and even opposite roles in breast cancer cell growth and metastasis. However, it remains unknown how the activities of various PI3K and AKT isoforms are coordinated during breast cancer progression. Previously, we showed WDR26 is a novel WD40 protein that binds Gβγ and promotes Gβγ signaling. Here, we demonstrate that WDR26 is overexpressed in highly malignant breast tumor cell lines and human breast cancer samples, and that WDR26 overexpression correlates with shortened survival of breast cancer patients. In highly malignant cell lines (MDA-MB231, DU4475 and BT549), downregulation of WDR26 expression selectively alleviated GPCR- but not EGF receptor-stimulated PI3K/AKT signaling and tumor cell growth, migration and invasion. In contrast, in a less malignant cell line (MCF7), WDR26 overexpression had the opposite effect. Additional studies indicate that downstream of GPCR stimulation, WDR26 serves as a scaffold that fosters assembly of a specific signaling complex consisting of Gβγ, PI3Kβ and AKT2. In an orthotopic xenograft mouse model of breast cancer, disrupting formation of this complex, by overexpressing WDR26 mutants in MDA-MB231 cells, abrogated PI3K/AKT activation and tumor cell growth and metastasis. Together, our results identify a novel mechanism regulating GPCR-dependent activation of the PI3K/AKT signaling axis in breast tumor cells, and pinpoint WDR26 as a potential therapeutic target for breast cancer.

## INTRODUCTION

The PI3Ks/AKTs are major effectors downstream of cell surface receptors, such as receptor tyrosine kinases (RTKs) and GPCRs [1, 2]. Dysregulation of the PI3K/AKT pathway occurs in more than 70% of breast cancers [3]. This pathway may be aberrantly activated by mutation or amplification of genes encoding PI3Ks or AKTs, loss or mutation of tumor suppressor PTEN, or mutation or overexpression of upstream signaling molecules such as HER2 or GPCRs. The class I PI3Ks consist of four isoforms, PI3Kα, PI3Kβ, PI3Kδ, and PI3Kγ [1]. These are obligate heterodimeric proteins composed of a catalytic (p110) subunit and one of several regulatory subunits, including p85. PI3Kα is predominantly activated by RTKs, and was the focus of many studies because its catalytic subunit (p110α) shows a high mutation rate (about 26%) in cancerous breast tumors [1]. PI3Kβ can be activated by RTKs or downstream of GPCRs through direct interaction with heterotrimeric G protein βγ subunits [4]. In cancers, no mutations in the p110β catalytic subunit of PI3Kβ have been identified, but in higher grade breast cancer tumors, HER2 expression and a poor prognosis has been correlated with overexpression of p110β [5]. Also, in transgenic mice, a catalytically inactive p110β inhibits HER2-driven tumor development [6]. In addition, PTEN-deficient breast tumor cells require PI3Kβ (but not PI3Kα) to proliferate, and are more sensitive to cell-cycle arrest induced by PI3Kβ inhibitors [7, 8]. PI3Kβ can be activated by RTKs but, in PTEN-deficient breast and prostate cancer cells, PI3Kβ appears to be activated primarily by GPCRs through Gβγ [8, 9]. These findings indicate that under certain circumstances, a Gβγ-stimulated PI3Kβ pathway may drive breast tumor progression. However, it remains to be determined how Gβγ specifically activates PI3Kβ in cancer cells, and how the PI3Kβ pathway is regulated during breast cancer progression.

AKT assumes three isoforms: AKT1, AKT2 and AKT3. Although these share a high sequence homology, different AKT isoforms have distinct functions in breast cancer progression [10, 11]. For example, although both AKT1 and AKT2 are required for primary tumor growth, AKT2 appears to promote tumor metastasis, while AKT1 has an inhibitory role in tumor invasion and metastasis [12, 13]. However, it is unclear how various AKT isoforms are activated by different PI3K isoforms and how they perform distinct functions in breast cancer.

GPCRs transmit extracellular signals and are the largest family of cell surface receptors. Experimental and clinical data indicate that breast cancer cells overexpress diverse GPCRs, such as lysophosphatidic acid (LPA) receptors, chemokine receptors (such as CXCR4 and CXCR7), and protease-activated receptors (PARs), and produce high levels of GPCR ligands (such as LPA and SDF1α) [3, 14–17]. Aberrant activation of GPCRs stimulates cancer cells not only to proliferate in primary sites but also to migrate and form metastasis in distant organs. We and others have shown that multiple GPCRs that promote breast tumor progression use Gβγ as a point of signal convergence [18, 19]. Blocking Gβγ signaling in cancer cells not only inhibited breast tumor cell growth but also dramatically blocked spontaneous lung metastasis in nude mice, suggesting that Gβγ may represent a novel therapeutic target for breast cancer [19]. Upon activation, Gβγ subunits regulate the activity of a diverse set of downstream signaling targets, e.g., PI3Ks, ERKs, and Rho guanine nucleotide exchange factors (RhoGEFs) for Rac and Cdc42 [20]. Still, it remains to be defined which downstream pathways mediate the function of Gβγ in breast cancer cells.

We recently identified WDR26 as a novel Gβγ-interacting protein that regulates the efficiency of Gβγ signaling [21, 22]. WDR26 is a member of the WD40-repeat proteins, which primarily function as scaffolds/adaptors, which coordinate formation of large and dynamic multi-protein complexes to orchestrate signaling networks [21, 23]. In leukemia cells, we first showed WDR26 is required for chemokine-induced cell migration [22]. It exists as a highly ordered oligomer that promotes specific and efficient chemokine-stimulated Gβγ signaling by coordinating Gβγ interaction with specific effectors, such as PLCβ2 [24]. These findings prompted us to ask whether WDR26 expression is perturbed in breast cancer and if it contributes to breast cancer progression. Our data showed that WDR26 is indeed upregulated in highly malignant breast tumors, and upregulated WDR26 promotes breast tumor growth and metastasis by acting as a scaffold that fosters Gβγ-mediated PI3K/AKT activation. These results have identified WDR26 as a novel regulator of the PI3K/AKT pathway and suggest that it may be a new prognostic marker and drug target in breast cancer.

## RESULTS

### WDR26 is upregulated in human breast cancer samples

**Figure 1:**
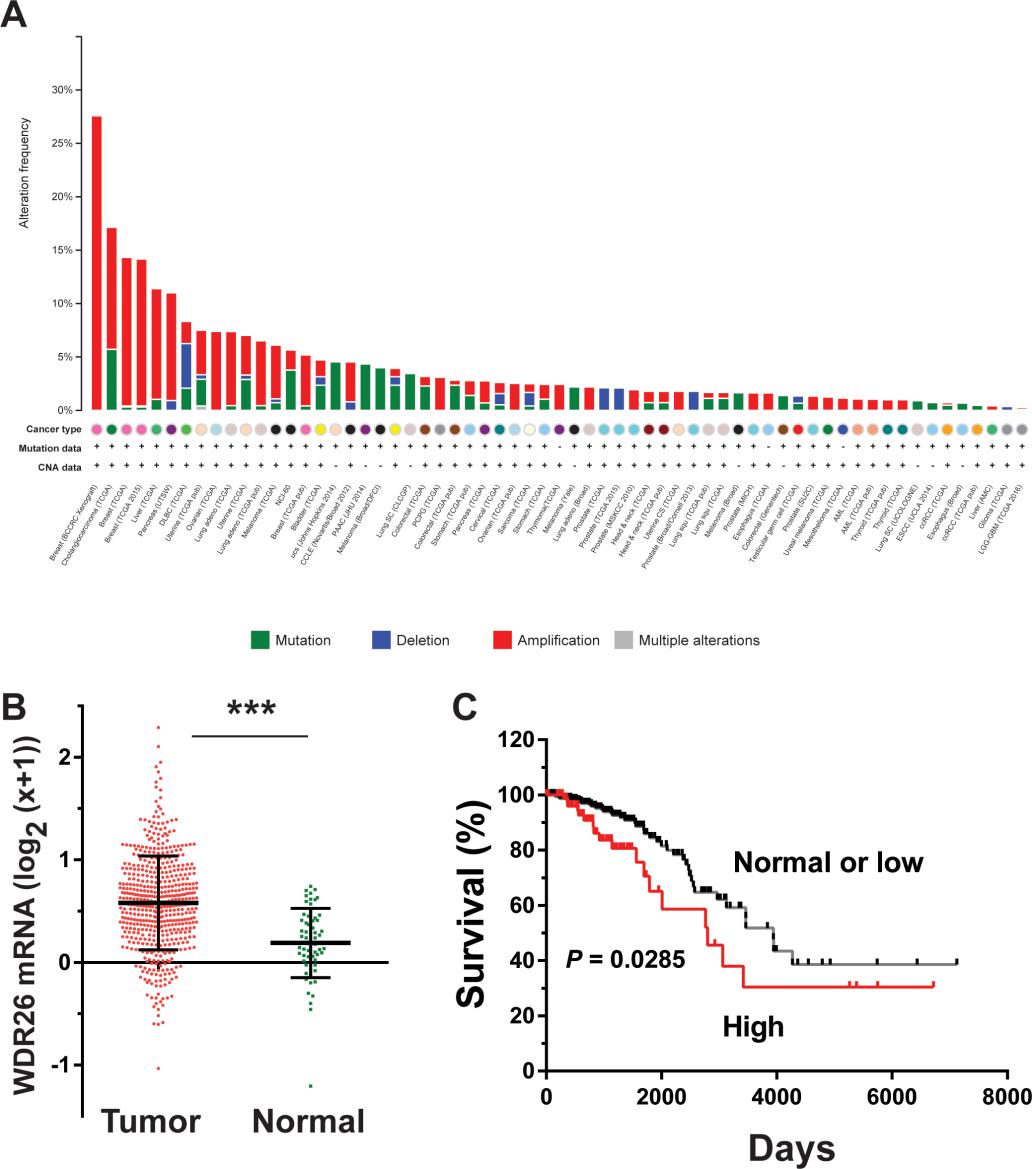
WDR26 expression is upregulated in breast cancer and correlated with poor patient survival **A.** breast cancer contains the highest frequency of WDR26 gene amplification. WDR26 genomic DNA copy-number variation across all tumor samples from 121 cancer studies was analyzed by cBioportal. **B.** WDR26 expression in TCGA invasive breast cancer dataset (n=63 for normal and n=534 for invasive cancer). *** p<0.0001. **C.** Kaplan-Meier curve shows the overall survival between samples overexpressing WDR26 (n=89) and samples expressing normal or low levels of WDR26 (n=368).

**Figure 2:**
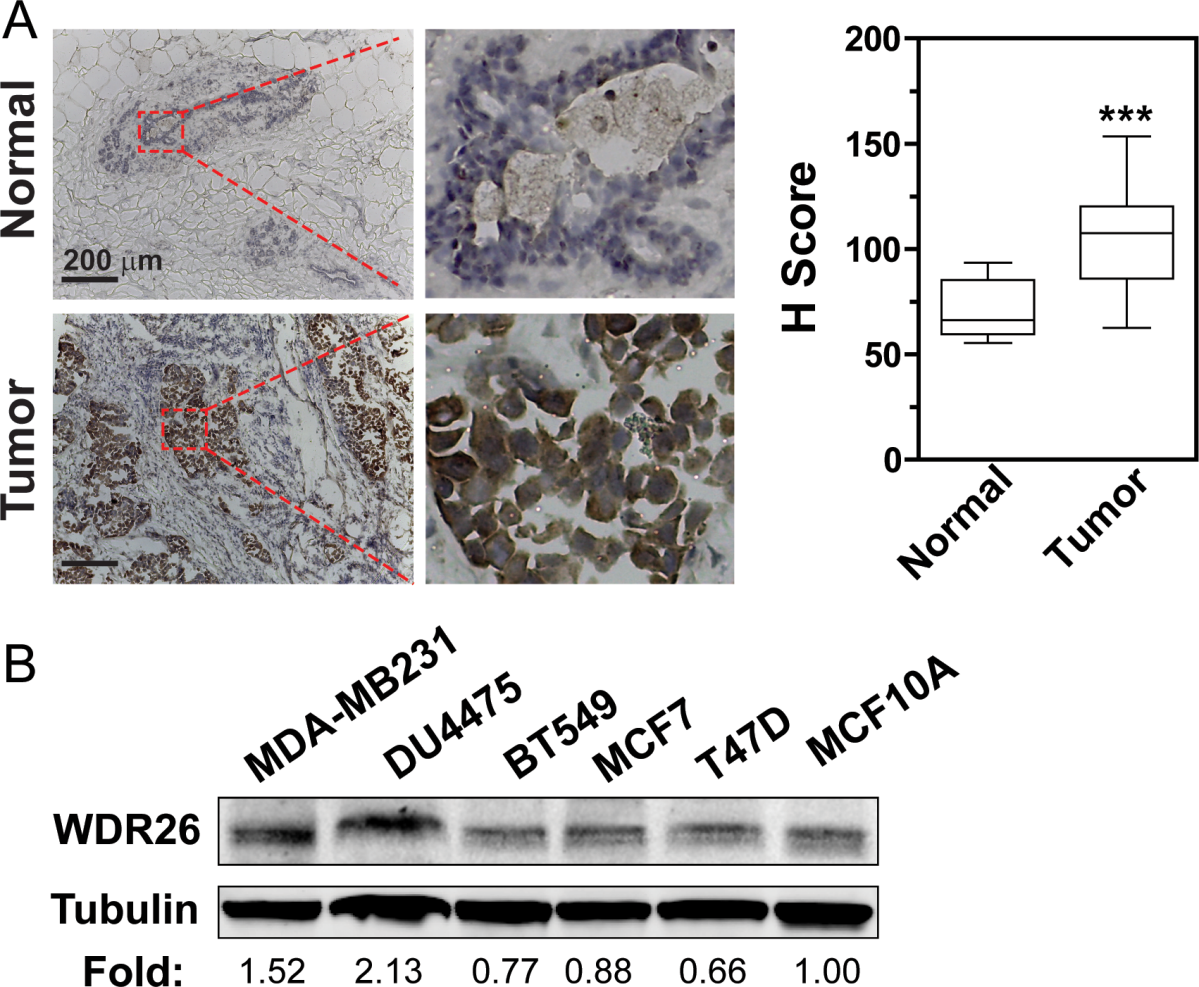
WDR26 is overexpressed in cell lines and tissue samples of human breast cancer **A.** WDR26 expression is significantly increased in malignant human breast cancer samples, as compared to normal breast tissues. Representative images show immunohistochemical staining of WDR26 protein in human breast cancer samples (Stage III) and matched, adjacent, normal tissues. Quantitative data are shown on the right. *** p<0.001 (n=15). **B.** Western blot analysis of WDR26 expression in human breast cancer cell lines. The relative level of WDR26 expression is expressed as a fold change over that in MCF10A (after normalization by tubulin) and indicated underneath the blot (n=3).

Analysis of a large cancer dataset compiled from 121 cancer studies by the cBioPortal showed that breast cancers had the greatest number of genomic copies of WDR26 (Figure 1A); and the basal-like breast cancer subtype had the highest frequency (27%) of WDR26 gene amplification, as compared to the luminal A (23%), luminal B (18%) and HER2 positive (16%) subtypes. Compared to normal breast tissues, breast cancer cells (from the TCGA invasive dataset, Cell, 2015) expressed significantly more WDR26 mRNA (Figure 1B). Importantly, WDR26 overexpression correlated with shorter patient survival (Figure 1C), suggesting WDR26 may be an important regulator or prognostic marker of breast cancer progression. Increased WDR26 expression in samples of malignant cancers at the protein level was verified immunohistochemically by comparing breast cancer tissues (stage II and III) to matched, adjacent, normal breast tissues (Figure 2A).

### WDR26 promotes breast cancer cell growth, migration and invasion

**Figure 3:**
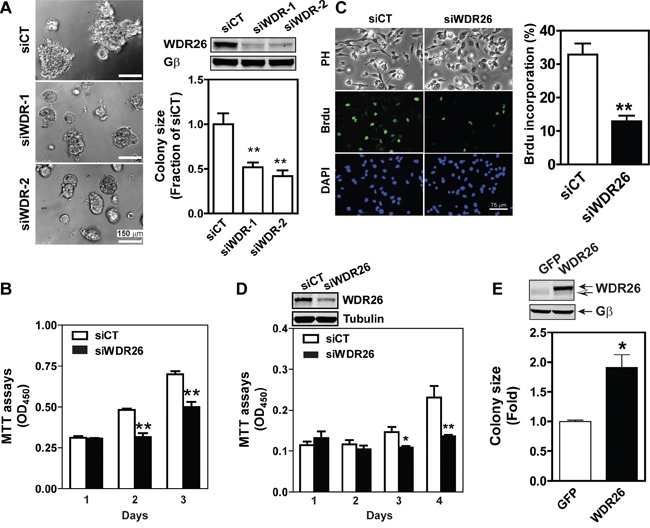
WDR26 promotes proliferation of breast cancer cells **A-D.** Downregulation of WDR26 inhibited breast cancer cell growth. MDA-MB231 (A-C) or DU4475 (D) cells were transiently transfected with a control (siCT) siRNA or siRNAs targeting two distinct regions of WDR26 (siWDR-1 and siWDR-2) (A) or siWDR-1 (siWDR26) (B-D). The effect on cell growth in Matrigel (A) was determined by phase-contrast imaging, followed by quantification of the size of the colonies. Colony size is expressed as the fraction of that derived from siCT-transfected cells. The effect on cell growth in 2D culture was measured by MTT assays (B and D) or BrdU incorporation assays (C). BrdU incorporation was detected by immunofluorescence staining. Phase contrast (PH) images of cells and fluorescence images of BrdU and nuclear (DAPI) staining are shown in (C) Quantitative data showing the level of BrdU incorporation are expressed as the percentage of cells stained with BrdU. *, ** p<0.05 and 0.01 *versus* siCT, respectively (n=5). *Insert*, representative blots showing WDR26 downregulation. **E.** overexpression of WDR26 promotes MCF7 cell growth. MCF7 cells stably expressing inducible GFP or WDR26 were treated with doxycycline (1 μg/ml) and cultured in Matrigel for ten days. The size of cell colonies was quantified and expressed as a fold-increase over that of GFP-expressing colonies. * p<0.05 *versus* GFP (n=4). *Insert*, a representative blot showing the level of WDR26 overexpression. Solid and hollow arrows indicate the ectopic and endogenous WDR26 expression in MCF7 cells, respectively.

To explore how WDR26 regulates the function of breast cancer cells, several cancer cell lines were screened for its expression. Compared to non-transformed MC10A cells, in MDA-MB231 and DU4475 cells (two highly metastatic breast cancer cell lines) WDR26 was overexpressed by 1.5 to 2 fold (Figure 2B). Furthermore, downregulating WDR26 levels by siRNAs (targeting two distinct coding regions of WDR26) reduced the size of MDA-MB231 cell colonies in Matrigel by 50%, demonstrating that WDR26 promotes breast cancer cell proliferation (Figure 3A). The decreased growth of WDR26-deficient cells was also shown by MTT-based cell viability assay and a 5-bromo-2′-deoxyuridine (Brdu) incorporation assay (Figure 3B-3C). Similar results were found for DU4475 and BT549 cells (Figure 3D and data not shown).

**Figure 4:**
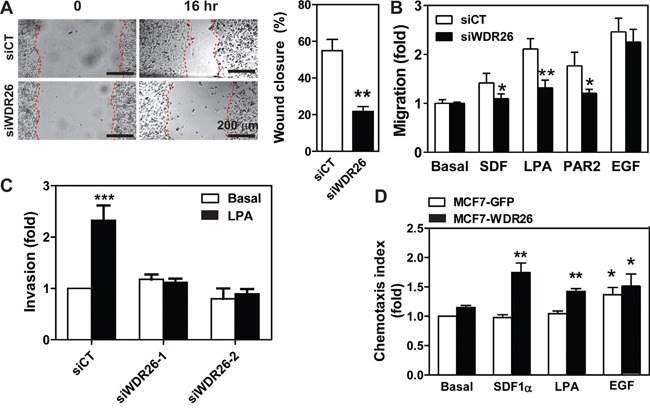
WDR26 promotes breast cancer cell migration and invasion **A-C.** MDA-MB231 cells were transiently transfected with a control siRNA (siCT) or siRNAs against WDR26 (siWDR26, siWDR26-1 or siWDR26-2). The effects on cell migration were determined by a wound healing assay (A) and transwell migration assay (B) in response to buffer (basal) or chemoattractants, SDF1α (100 nM), LPA (50 nM), PAR2 agonist peptide (10 μM) and EGF (50 ng/ml). The effect of LPA on cell invasion was determined in a transwell migration assay with the filter coated with a thin layer of Matrigel (C). *, **, *** p<0.05, 0.01 and 0.001, respectively, *versus* siCT (n=4). **D.** the effect of WDR26 overexpression on MCF7 cell migration. MCF7 cells stably expressing inducible GFP or WDR26 were treated with doxycycline (1 μg/ml) for 5 days and then subjected to the transwell migration assay as described in B. *, ** p<0.05 and 0.01, respectively, *versus* basal (n=4).

Migration of MDA-MB231 cells was also inhibited by downregulation of WDR26, as shown in a wound-healing assay (Figure 4A). It is unlikely that cell migration decreased because of changes in cell growth, since the assay was performed in the presence of the cell proliferation inhibitor, mitomycin. Notably, cell migration was blocked by WDR26 knockdown in a transwell-based assay if the chemoattractants were GPCR ligands (i.e., SDF1α, LPA and a PAR2 peptide agonist) but not growth factors, such as EGF (Figure 4B). Invasion of MDA-MB231 cells into Matrigel can also be induced by LPA; and this migratory behavior was also blocked by WDR26 downregulation (Figure 4C). Similarly, WDR26 inhibition significantly reduced GPCR-stimulated BT549 cell migration (data not shown).

To confirm further that WDR26 plays an important role in breast cancer cell growth, migration and invasion, we examined the effects of ectopic WDR26 expression in MCF7 cells, which only express a low level of endogenous WDR26. WDR26 upregulation significantly increased the size of MCF7 cell colonies grown in Matrigel by 2 fold (Figure 3E), and enhanced cell migration induced by LPA and SDF1α by 1.5 to 1.8 fold, respectively (Figure 4D). In contrast, EGF-induced cell migration was not significantly affected by WDR26 overexpression (Figure 4D). Together, these findings indicate that WDR26 selectively promotes GPCR-stimulated breast cancer cell growth, migration and invasion.

### WDR26 selectively regulates GPCR-mediated PI3K/AKT activation

**Figure 5:**
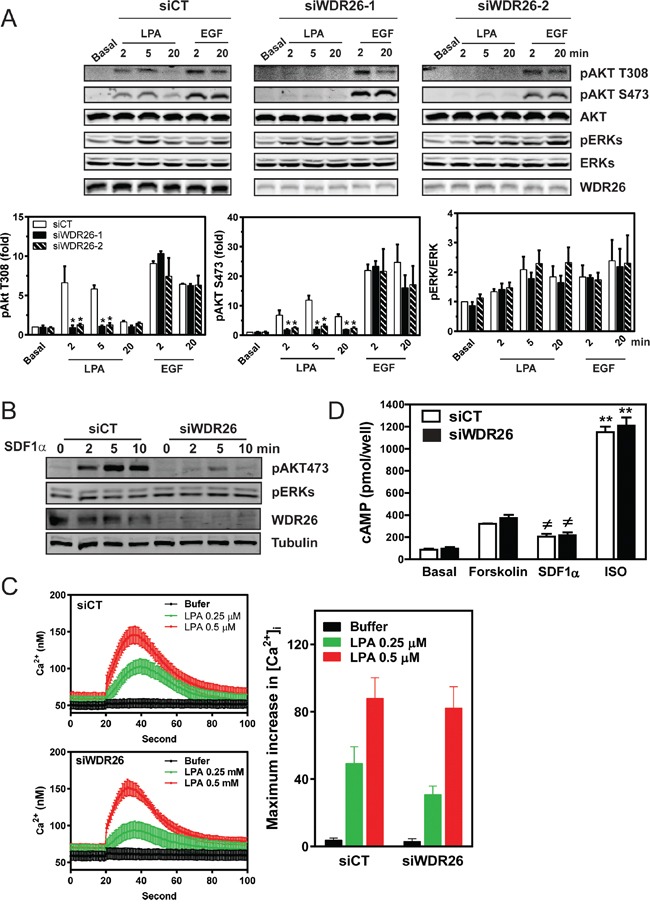
WDR26 selectively regulates Gβγ-mediated AKT phosphorylation MDA-MB231 cells were transiently transfected with a control siRNA (siCT) or siRNAs targeting WDR26 (siWDR26), and the effects on Gβγ-AKT phosphorylation and Gα-mediated cAMP accumulation or Ca^2+^ signaling were determined. **A-B.** LPA (10 μM)-, EGF (50 ng/ml) (A)- or SDF1α (100 nM)(B)-stimulated AKT and ERK phosphorylation determined by Western blotting. Data in A are expressed as the fold-increase over the unstimulated control (basal) after the levels of phosphorylated proteins are normalized to that of total proteins. *, ** p<0.05, *versus* siCT (n=5). **C-D.** LPA-stimulated Ca^2+^ signaling (C), SDF1α-mediated decrease in cAMP accumulation and isoproterenol (ISO)-stimulated increase in cAMP (D). Ca^2+^ signaling was measured using Fura-2/AM. Representative data and maximum Ca^2+^ concentration (n=3) are shown in C. To measure SDF1α- and ISO-induced decrease and incrase in cAMP accumulation, respectively, cells were treated with either forskolin (10 μM) alone or forskolin (10 μM) plus SDF1α (50 nM) or ISO (1 μM) (n=3).

**Figure 6:**
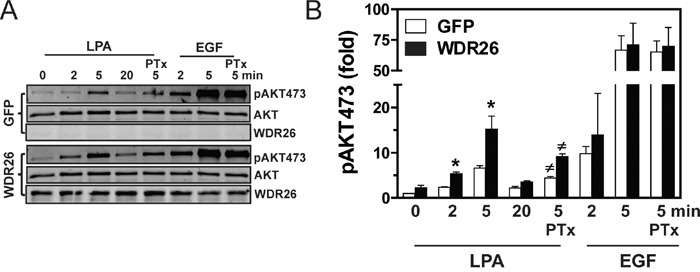
Overexpression of WDR26 in MCF7 cells enhances PI3K/AKT activation **A.** representative blots show AKT phosphorylation in MCF7 cells expressing inducible GFP or WDR26. To determine the effect of PTx, cells were pre-treated with PTx (200 ng/ml) overnight. **B.** Quantitative data from three independent experiments. Data are expressed as a fold-increase over GFP basal. * p<0.05 *versus* GFP basal; # p<0.05 *versus* PTx-untreated.

To probe how WDR26 regulates GPCR signaling in breast cancers, we examined the effect of WDR26 downregulation on the activation of Gα- or Gβγ-mediated signaling pathways downstream of GPCR stimulation. Previously, we showed that, in MDA-MB231 cells, LPA and SDF1α stimulate AKT and ERK phosphorylation, primarily through Gβγ [19]. WDR26 knockdown reduced the level of AKT phosphorylation by 2 to 6 fold at different time points that is stimulated by LPA or SDF1α, but had little effect on ERK phosphorylation (Figure 5A-5B). Moreover, WDR26 downregulation did not affect LPA stimulated Ca^2+^ signaling or a SDF1α-stimulated decrease in cAMP accumulation (Figure 5C-5D), which are mediated by the activation of Gαq/11 and Gαi/o subunits, respectively [22, 25]. Additionally, WDR26 downregulation did not affect isoproterenol-stimulated cAMP accumulation, which is mediated by the activation of Gαs downstream of β-adrenergic receptors (Figure 5D). These findings indicate that WDR26 selectively regulates Gβγ-mediated PI3K/AKT activation. Consistent with a specific role for WDR26 in regulating GPCR signaling, suppressing WDR26 did not affect EGF-stimulated AKT or ERK phosphorylation (Figure 5A). Similarly, in BT549 cells, suppressing WDR26 selectively alleviated LPA- and SDF1α-, but not EGF-stimulated AKT phosphorylation (data not shown). Conversely, in MCF7 cells, overexpressing WDR26 selectively enhanced LPA- but not EGF-stimulated AKT phosphorylation by 2 to 3 fold (Figure 6A-6B). Notably, in both control and WDR26-overexpressing MCF7 cells, pertussis toxin inhibited LPA-stimulated AKT phosphorylation, suggesting that Gβγ released from activated Gi/o proteins stimulates the phosphorylation of AKT (Figure 6A-6B).

**Figure 7:**
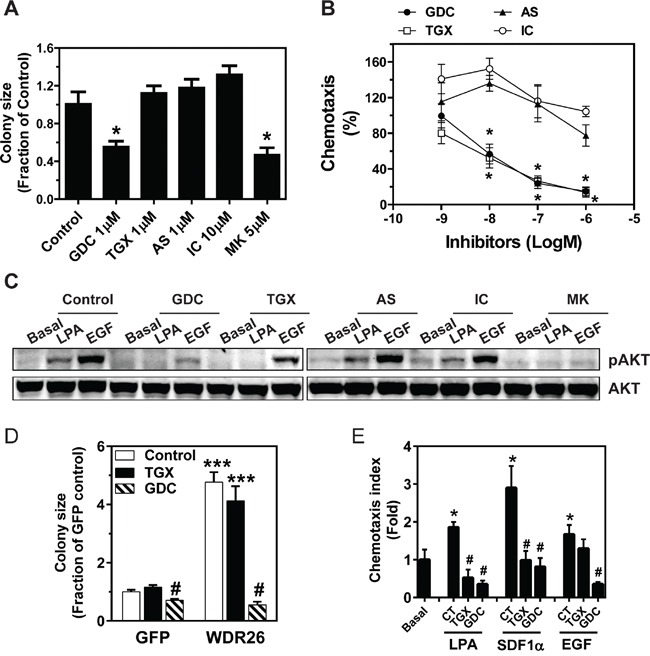
PI3K/AKT activation contributes to WDR26-regulated breast cancer cell growth and migration **A-C.** effects of PI3K and AKT inhibitors on MDA-MB231 cell growth and migration. MDA-MB231 cells were treated with the indicated PI3K and AKT inhibitors at the indicated doses, and the effects on tumor cell growth in Matrigel (A), cell migration (B) and LPA- and EGF-simulated AKT phosphorylation (C) were determined. *p<0.05 *versus* untreated control (n=3). GDC(GDC0941), TGX(TGX221), AS(AS605240), IC(IC87114) and MK(MK2206) are inhibitors for pan-PI3K, PI3Kβ, PI3Kγ, PI3Kδ and pan-AKT, respectively. **D-E.** effects of PI3K inhibitors on the growth (D) and migration (E) of MCF7 cells overexpresssing GFP or WDR26. TGX, TGX221 1μM; GDC, GDC0941 1 μM; CT, control. *** p<0.001 *versus* GFP control; ^#^p<0.01 *versus* GFP or WDR26 control (n=3) in D. * p<0.05 *versus* basal; ^#^ p<0.01 *versus* CT (n=4) in E.

To test whether the PI3K/AKT pathway contributes to breast cancer cell growth and migration, the effects of PI3K inhibitors on MDA-MB231 cells were examined. No effect was seen after treating cells with PI3K isoform-selective inhibitors (TGX221, AS605240 and IC87114 for p110β, p110γ and p110δ, respectively) at doses known to discriminate different isoforms, whereas, MDA-MB231 growth in Matrigel was partly inhibited by the pan-PI3K inhibitor, GDC0941, or pan-AKT inhibitor, MK2206, suggesting several isoforms of PI3K and AKT work in concert to drive breast cancer cell growth (Figure 7A). In contrast, LPA-mediated transwell migration was sensitive to both TGX221 and GDC0941, but not AS605240 and IC87114, suggesting the specific involvement of PI3Kβ in cell migration (Figure 7B). Consistent with an isoform-specific effect on PI3K-mediated AKT activation, TGX221 completely abolished LPA-stimulated AKT phosphorylation, which is known to involve PI3Kβ, but only partially inhibited EGF-simulated AKT phosphorylation, which is mediated primarily via PI3Kα (Figure 7C). In contrast, neither LPA- nor EGF-induced AKT phosphorylation was affected by the PI3Kδ- and PI3Kγ-specific inhibitors, although they were sensitive to the pan-PI3K and pan-AKT inhibitors (Figure 7C).

To examine how changes in PI3K activation might contribute to WDR26-regulated breast cancer cell growth and migration, we determined the effect of specific PI3K inhibitors on the enhanced MCF7 cell growth and migration induced by WDR26 overexpression. Treatment with the PI3Kβ inhibitor, TGX221, had no effect on the growth of MCF7 cells expressing GFP or WDR26 (Figure 7D). However, treatment with the pan-PI3K inhibitor, GDC0941, completely alleviated the increased MCF7 cell growth induced by WDR26 overexpression (Figure 7D). In contrast, LPA- and SDF1α-induced migration in WDR26-overexpressing MCF7 cells was sensitive to both TGX221 and GDC0941, while EGF-stimulated cell migration was sensitive to GDC0941 but not TGX221 (Figure 7E). Together, these findings indicate that WDR26 promotes breast cancer growth and migration primarily through the PI3K/AKT pathway.

### WDR26 fosters the interaction between Gβγ, PI3Kβ and AKT2 to promote PI3K/AKT activation

**Figure 8:**
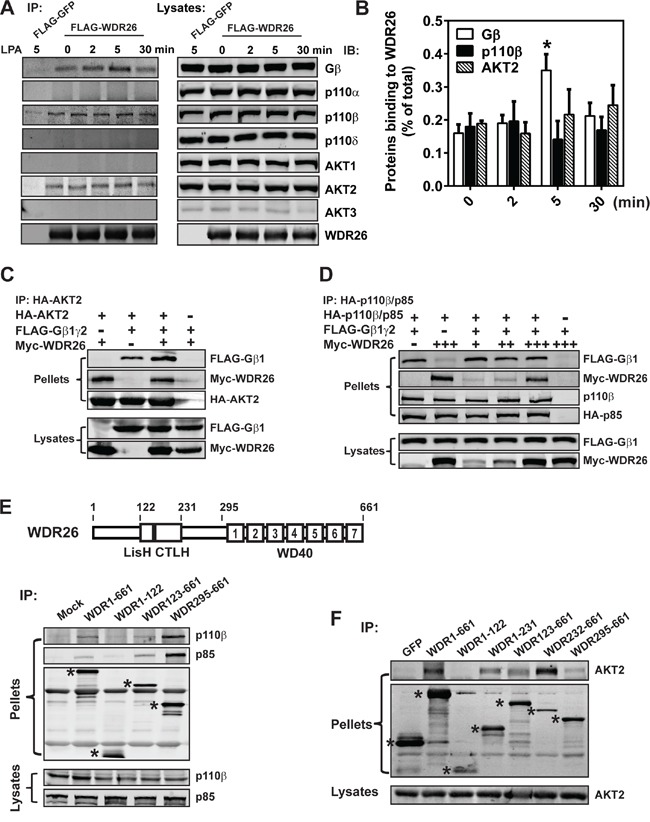
WDR26 fosters the interactions between Gβγ, PI3Kβ and AKT2 **A-B.** WDR26 is selectively co-immunoprecipitated with endogenous Gβγ, PI3Kβ and AKT2 in MDA-MB231 cells. MDA-MB231 cells expressing FLAG-GFP or FLAG-WDR26 were stimulated with LPA (10 μM) for the indicated time and subjected to immunoprecipitation with an anti-FLAG antibody. Co-immunoprecipitated proteins were detected with the indicated antibodies. Representative blots are shown in A and quantitative data from three independent experiments are shown in (B) The amount of immunoprecipitated proteins is expressed as the percentage of total proteins in the lysates, with the background binding from FLAG-GFP immunoprecipitates substracted. * p<0.05 *versus* 0 min (n=3). **C.** WDR26 binds AKT2 and enhances its interaction with Gβγ. HEK293 cells were transiently transfected with HA-AKT2 and FLAG-Gβ1γ2 or Myc-WDR26, or FLAG-Gβ1γ2 plus Myc-WDR26. Cell lysates were immunoprecipitated with an anti-HA antibody. **D.** WDR26 binds PI3Kβ and forms a trimeric complex with PI3Kβ and Gβ1γ2. MDA-MB231 cells transduced with adenoviruses encoding the indicated proteins were subjected to co-immunoprecipitation assays as described in (C) **E-F.** the binding sites of PI3Kβ and AKT2 on WDR26. FLAG-GFP, FLAG-WDR26 and its deletion mutants were transiently co-expressed with PI3Kβ in MDA-MB231 (E) or AKT2 in HEK293 (F) cells, and were immunoprecipitated from cell lysates with an anti-FLAG antibody. The bands corresponding to GFP, WDR26, and its mutants are indicated by asterisks. A schematic representation of the WDR26 structure is shown in the top panel of E. Experiments in (C-F) were repeated for at least three times with similar results.

Previously, we reported that in leukemia cells, WDR26 fosters the interaction between Gβγ and its effector PLCβ2 by directly binding both proteins, thereby promoting Gβγ-stimulated PLCβ2 activation and Ca^2+^ signaling [24]. We therefore postulated that WDR26 similarly regulates PI3K/AKT activation in breast cancer cells by fostering the interactions between Gβγ, PI3K and AKT. To test this, FLAG-tagged WDR26 was overexpressed in MDA-MB231 cells and complexes were co-immunoprecipitated with an anti-FLAG antibody (because no commercially available antibodies can efficiently immunoprecipitate endogenous WDR26). WDR26 was found to be associated with Gβγ in unstimulated cells (Figure 8A-8B). Upon stimulation with LPA, the association of WDR26 with Gβγ increased by 2 fold within 5 minutes; and then, after prolonged stimulation for 30 minutes, the association decreased to a basal level. WDR26 also interacted with the PI3K subunit, p110β, and AKT2 in unstimulated cells. In contrast, LPA stimulation did not affect the interaction of p110β and AKT2 with WDR26. Notably, although MDA-MB231 cells express other isoforms of PI3Ks and AKT (including p110α and p110δ, and AKT1), these did not associate with WDR26 in either stimulated or unstimulated cells (Figure 8A), suggesting that WDR26 selectively interacts with specific isoforms of PI3Ks and AKTs.

The interactions between WDR26 and AKT2 and PI3Kβ were examined further by co-immunoprecipitation assays to assess how they might influence formation of a signaling complex containing WDR26, Gβγ, PI3Kβ and AKT2. As shown in Figure 8C-8D, AKT2 and PI3Kβ interacted individually with WDR26 and Gβγ, and also formed a trimeric complex with WDR26 and Gβγ. The presence of WDR26 did not affect Gβγ interaction with PI3Kβ but enhanced Gβγ interaction with AKT2. Given that WDR26 also binds Gβγ, these findings indicate that WDR26 likely serves as a scaffold that fosters interactions between Gβγ, PI3Kβ and/or AKT2, thereby promoting PI3Kβ and AKT2 activation by Gβγ.

To identify PI3Kβ and AKT2 binding sites on WDR26, deletion mutants of FLAG-tagged WDR26 were co-expressed with HA-p110β and HA-p85 or HA-AKT2 and protein complexes were co-immunoprecipitated. A truncation mutant lacking the WDR26 N terminal, and consisting of only the LisH-CTLH and WD40 domains, appeared to bind both PI3Kβ and AKT2, while WDR1-122, the N-terminal fragment, did not (Figure 8E-8F). The WDR295-661 mutant, which lacks the N-terminal half and LisH-CTLH domain but retains the WD40 repeats, bound both p110β/p85 and AKT2; however, the binding sites for AKT2 may involve other domains because WDR232-661 bound AKT2 better than WDR295-661, and WDR1-231 also interacted with AKT2 (Figure 8F).

**Figure 9:**
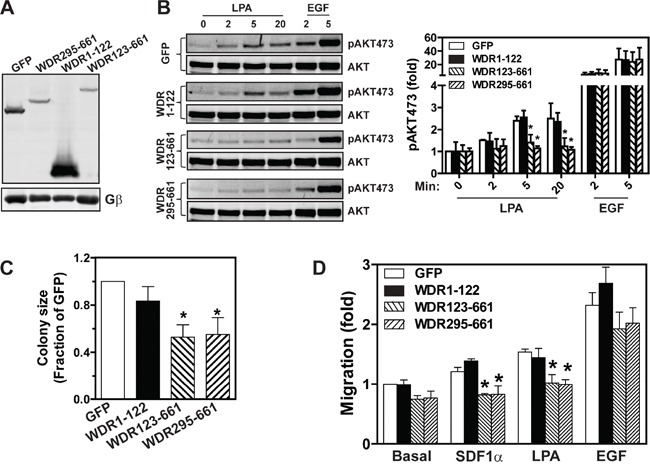
The effects of WDR26 mutants on AKT activation, tumor cell growth, and migration **A.** Western blot analysis shows expression of GFP and WDR26 mutants in MDA-MB231 cells induced by a three-day treatment with doxycycline. **B.** LPA- and EGF-stimulated AKT phosphorylation in MDA-MB231 cells expressing the indicated constructs (n=4). **C-D.** effects of overexpressing WDR26 mutants on MDA-MB231 cell growth in Matrigel (C) and migration (D). * p<0.05 *versus* GFP (n=4).

Previously, we showed that the WDR123-661 deletion mutant can bind both Gβγ and its effector, PLCβ2 [24]. However, when it was overexpressed in leukocytes, it acted as a dominant-negative mutant: it inhibited full-length WDR26 from promoting Gβγ-mediated Ca^2+^ signaling and cell migration [24]. Given that the WDR26 fragments, WDR123-661 and WDR295-661, could still interact with AKT2 and PI3Kβ, we reasoned that they might also interfere with the function of the full-length WDR26 in breast cancer cells. To test this, we generated stable MDA-MB231 cells expressing inducible WDR123-661 and WDR295-661, with GFP and WDR1-122 as controls. As compared to GFP, induced expression of WDR1-122 had no effect on LPA- or EGF-stimulated AKT phosphorylation (Figure 9A-9B). In contrast, inducing expression of WDR123-661 and WDR295-661 in MDA-MB231 cells decreased LPA-stimulated AKT phosphorylation by 50% (Figure 9A-9B). Consistent with their effect on AKT activation, induced expression of WDR123-661 and WDR295-661, but not WDR1-122, significantly reduced the size of MDA-MB231 cell colonies in Matrigel (Figure 9C), and LPA- or SDF1α-induced cell migration (Figure 9D). The effect of these mutants is likely to be specific since WDR123-661 and WDR295-661 neither affected EGF-stimulated AKT activation nor cell migration (Figure 9B and 9D).

### WDR26 promotes breast tumor growth and metastasis *in vivo*

**Figure 10:**
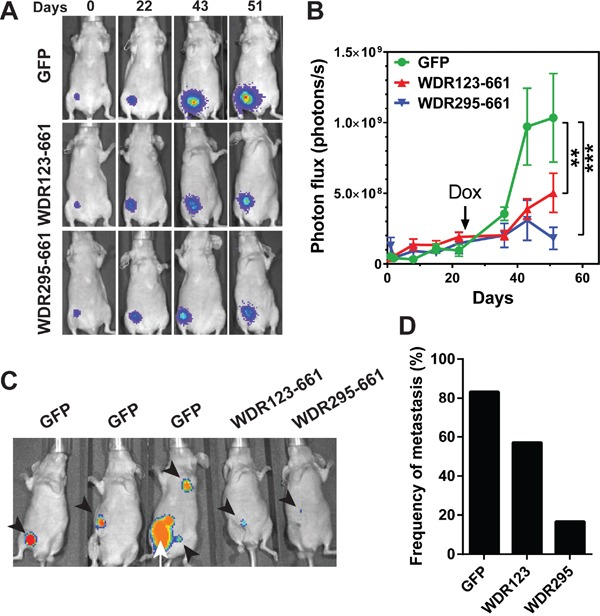
WDR26 mutants inhibits tumor growth and metastasis in mice MDA-MB231 cells expressing luciferase and inducible GFP or WDR26 mutants were implanted into the inguinal mammary fad pads of nude mice (n=6-7). 23 days after implantation, mice were treated with doxycycline to induce the expression of GFP and WDR26 mutants. Tumor growth was monitored by bioluminescence imaging. Primary tumors were resected when they reached a diameter of ~500 mm^3^, and tumor metastases were detected 4 weeks after surgery. **A-B.** representative bioluminescence images (A) and quantitative data showing primary tumor growth at the indicated times. **, *** p<0.01 and 0.001, respectively *versus* GFP. **C.** representative images showing the sites of metastases in mice transplanted with MDA-MB231 cells expressing GFP or WDR26 mutants. Arrowheads indicate metastases; arrows indicate the recurrence of primary tumor. **D.** the frequency of metastases in mice transplanted with MDA-MB231 cells expressing GFP (83%) or WDR26 mutants, WDR123-661 (WDR123; 57%) and WDR295-661 (WDR295; 18%).

To investigate whether WDR26 promotes breast cancer cell growth and metastasis *in vivo*, we inoculated equal numbers of MDA-MB-231 cells that expressed inducible GFP, WDR123-661, or WDR295-661 into the mammary fat pad of nude mice, and allowed them to form palpable tumors without inducing protein expression for 23 days. Over this period, the growth rate of cells expressing GFP, WDR123-661 or WDR295-661 was comparable, as determined by BLI (Figure 10A-10B); however, 10 days after the transgenes were induced by doxycycline, WDR123-661 and WDR295-661-expressing cells formed significantly smaller tumors than GFP-expressing cells (Figure 10A-10B). These data indicate that WDR26-mediated signaling is critical for the progression of established MDA-MB-231 tumor xenografts.

To determine the role of WDR26 in spontaneous tumor metastasis and exclude the possibility that tumor size affects metastasis formation, we resected primary tumors derived from GFP, WDR123-661 or WDR295-661-expressing cells when they reached the size of ~ 500 mm^3^, and monitored tumor metastasis for four additional weeks. Of seven mice bearing GFP-expressing tumors, one died in the cage before its tumor reached ~500 mm^3^, thus excluding it from examination for metastasis. Nevertheless, of the six remaining mice bearing GFP expressing-tumors, five (83%) developed metastases at multiple sites, including the local and distant lymph nodes (Figure 10C-10D). In contrast, only 4 of 7 (57%) and 1 of 6 (16%) mice bearing WDR123-661 and WDR295-661-expressing tumors, respectively, developed metastases; and these were smaller and primarily at local lymph nodes (Figure 10C-10D). These findings indicate that WDR26 is required for spontaneous metastasis of breast cancer cells.

## DISCUSSION

In this study, we identified WDR26 as a novel regulator of the PI3K/AKT pathway, and showed that aberrant expression of WDR26 in breast cancer cells contributes to breast cancer cell growth and metastasis. Our data indicate that WDR26 is upregulated in malignant cancer tissues and its expression strongly correlates with poor patient survival. Compared to less malignant and non-invasive cell lines, such as MCF7 and T47D, highly malignant and invasive breast cancer cell lines (i.e, MDA-MB231 and DU4475) expressed significantly more WDR26. Because we had access to only a limited number of clinical samples and cell lines, it remains to be determined whether WDR26 expression correlates with particular breast cancer subtypes or histological features. Nevertheless, our data demonstrate that WDR26 overexpression is functionally significant, since knockdown of WDR26 in MDA-MB231 and DU4475 cells inhibited growth, migration, and invasion, while overexpression of WDR26 in MCF7 cells had the opposite effect.

Our data argue that WDR26 promotes breast cancer growth and metastasis through the GPCR-mediated PI3K/AKT signaling pathway. In breast cancer cells, downregulation of WDR26 selectively impaired GPCRs from inducing AKT phosphorylation via Gβγ, but did not prevent RTKs from signaling for growth factor-mediated AKT activation. Moreover, the regulation of WDR26 on Gβγ signaling is highly specific, since downregulation of WDR26 affected neither LPA- nor SDF1α-stimulated ERK activation, despite that both PI3K/AKT and ERK are activated downstream of Gβγ. These findings are consistent with our previous report that WDR26 is a selective regulator of Gβγ-mediated signaling pathways downstream of GPCR activation [22].

Our results indicate that the specificity of WDR26 in regulating the Gβγ-mediated PI3K/AKT signaling likely stems from a specific interaction with multiple, key components of this pathway, and that this interaction tethers them to a signaling complex. Consistent with what we reported previously [22]. WDR26 interacts with Gβγ liberated from heterotrimeric G proteins by GPCR stimulation. WDR26 also binds Gβγ-specific PI3Kβ, but not RTK-specific PI3Ks, including PI3Kα and PI3Kδ. Additionally, WDR26 selectively binds AKT2 but not AKT1. As with Gβγ, WDR26 likely binds PI3Kβ and AKT2 directly, since their interaction can also be demonstrated *in vitro* using purified proteins (data not shown). Nevertheless, unlike Gβγ, the interaction of PI3Kβ and AKT2 with WDR26 was not enhanced by GPCR stimulation, suggesting that they likely exist in a preformed complex in unstimulated cells. The ability of WDR26 to interact with multiple proteins is consistent with its scaffolding function we reported previously [24], but the molecular basis for its binding to specific PI3K and AKT isoforms remains to be defined. Moreover, we are unable to narrow down the exact domains of WDR26 that bind Gβγ, PI3Kβ and AKT2. This might be because the WDR26 scaffold is shaped by a highly ordered oligomer and uses domains from different WDR26 monomers to bind certain proteins [24].

WDR26 might tether Gβγ, PI3Kβ, and AKT2 in a complex, allowing PI3Kβ and AKT2 to work in concert, and with specificity. PI3Kβ is activated by binding to Gβγ, but we found WDR26 was not required for the Gβγ/PI3Kβ interaction. Nevertheless, WDR26 may bring PI3Kβ in the vicinity of Gβγ for more efficient activation. Our results indicate Gβγ binds AKT2 and WDR26 enhances this interaction. Thus, by recruiting AKT2 to the signaling complex containing Gβγ and PI3Kβ, WDR26 likely enhances PI3Kβ specificity for certain AKT isoforms. Our results also suggest WDR26 integrates interactions between Gβγ, PI3Kβ, and AKT2, which is critical for Gβγ-mediated PI3K/AKT signaling and function in breast cancer cells. Thus, overexpression of WDR26 N-terminal truncation mutants, WDR123-661 and WDR295-661, blocks AKT activation and breast cancer cell growth and migration *in vitro* and *in vivo*. As we reported previously [24], full-length WDR26 is expected to oligomerize to form a scaffold, while the N-terminal truncation of WDR26 likely disrupts oligomerization. These truncation mutants, however, still bind Gβγ, PI3Kβ, and AKT2, and when overexpressed, they are expected to block the ability of full-length WDR26 to form the scaffold that facilitates PI3Kβ/AKT2 activation and function. This would explain why WDR26 mutants lacking N-terminal domains are dominant negative inhibitors of breast cancer growth and migration.

Accumulating evidence indicates that distinct PI3K and AKT isoforms play different roles in breast cancer progression [1, 10, 11]. In tumors with PI3Kα mutation or activated RTKs, PI3Kα drives tumor progression [27, 29]. In contrast, PTEN-deficient tumors are driven primarily by PI3Kβ [7, 8, 28]. However, recent studies indicate that inhibition of one PI3K isoform in breast cancer containing PTEN deficiency, PI3Kα mutation or HER2 amplification may lead to compensatory reactivation of another [29, 30]. Although both AKT1 and AKT2 are required for breast tumor growth, AKT1 appears to suppress, while AKT2 promotes tumor metastasis [12, 13, 31]. A recent study also showed that AKT2 but not AKT1 is required for the maintenance of PTEN-deficient breast tumor spheroids in 3D culture [32]. Interestingly, although WDR26 selectively regulates PI3Kβ and AKT2 signaling, its activity on breast cancer cell growth and migration does not seem to depend on the status of PI3Kα mutation, PTEN expression or RTK overexpression. For example, MDA-MB231 and DU4475 cells are triple-negative cell types that express wild-type PTEN, while PTEN is lost in BT549 cells. Nevertheless, they all display reduced growth and migration when WDR26 expression is inhibited. Similarly, although MCF7 cells contain mutations that activate PI3Kα, if they overexpress WDR26, then their growth and GPCR-induced cell migration accelerates. Nevertheless, the fact that WDR26 expression is not significantly upregulated in BT549 cells suggests that PTEN deficiency may render BT549 cells more dependent on the WDR26-regulated PI3K/AKT pathway. Notably, suppressing PI3Kβ activity alone does not block MDA-MB231 or WDR26-overexpression MCF7 cells from growth in Matrigel. These results suggest that, in promoting breast cancers, WDR26 must do more than simply augment PI3Kβ activity. Rather, it must also uniquely promote signal transduction from PI3Kβ specifically to AKT2-dependent pathways. Future work will test whether WDR26 modulates the activity of AKT2 selectively, and look to target WDR26 as a way to disrupt the PI3Kβ-AKT2 signaling axis. Such approaches may offer advantages over the inhibition of individual PI3K isoforms, which is expected to cause non-selective suppression of the activity of all AKT isoforms, and may also cause compensatory reactivation of others [33]. Since inhibiting WDR26 blocks breast cancer cell migration *in vitro* and metastasis *in vivo*, targeting WDR26 may also represent a novel approach for the treatment of both primary and metastatic tumors.

In summary, we demonstrated that WDR26 is overexpressed in highly malignant breast cancer cells and patient samples, and upregulated WDR26 overactivates the Gβγ-mediated PI3K/AKT pathway downstream of multiple GPCRs, promoting breast cancer cell growth, migration and invasion. Mechanistically, we showed WDR26 functions as a scaffolding protein to foster the interactions between Gβγ, PI3Kβ and AKT2, thereby promoting Gβγ-mediated PI3K/AKT activation. Together, our results define an unknown mechanism of regulating GPCR-dependent activation of the PI3K/AKT signaling axis in breast tumor cells, and identify WDR26 as a potential novel target against breast cancer.

## MATERIALS AND METHODS

### The cancer genome atlas data analyses

The cBioportal for Cancer Genomics (www.cbioportal.org/) was used to analyze WDR26 gene mutation and copy-number variations across all available 121 cancer datasets, and the association of WDR26 mRNA expression level with the overall survival (OS) in the breast invasive carcinoma dataset (TCGA, Cell, 2015) [26]. A two-fold z-score threshold was used to identify patients with the alternation of WDR26 mRNA expression levels. WDR26 mRNA expression data were transformed as log_2_ (x+1). The correlation of WDR26 expression and patient survival was analyzed according to the Kaplan-Meier method using the Graphpad Prism 6 Software.

### Immunohistochemistry

Frozen human breast tissues were purchased from the University of Massachusetts Cancer Center Tissue Bank. They were sectioned at 10 μm intervals and stained for WDR26 using the rabbit primary antibody against WDR26 (Bethyl laboratories, USA) (1:800) and the ultra-sensitive ABC peroxidase staining kit (Pierce) [19]. The specificity of WDR26 staining was determined by blocking the anti-WDR26 antibody with the antigen peptide overnight. The intensity of staining was evaluated semi-quantitatively using a relatively unbiased Computer Assisted Image Analysis approach [34], and expressed as the H score, which is defined as intensity of staining with a value of 0, 1 (+), 2 (++), 3 (+++) times the percentage of stained areas at each intensity.

### Western blotting analysis

Cells or tissues were lysed in the RIPA buffer (50 mM Tris-HCl, pH7.4, 150 mM NaCl, 1% Nonidet P-40, 0.1% SDS and 1 mM EDTA) containing protease and phosphatase inhibitors. Western blotting was performed as we reported [19].

### Lentiviral constructs and virus generation

Human WDR26 and mutants were amplified by PCR and cloned into the pSLIK lentiviral destination vector for Tet-inducible expression, using the Gateway cloning kit (Invitrogen) [19]. Lentivirus was generated as described previously [19].

### Cell culture and establishment of stable cell lines

All human breast carcinoma cell lines used here were from ATCC, maintained in standard media and used at low passages. The MDA-MB231and MCF7 cells stably expressing luciferase were transduced with pSLIK lentiviruses encoding Tet-inducible EGFP, WDR26, or WDR26 mutants, and selected with hygromycin (0.2-0.5 mg/ml) to establish stable cell lines.

### siRNA and transfection

The control and WDR26 siRNAs were described previously [22]. Transient transfection of breast cancer cells with siRNAs was performed using the Neon transfection device (Invitrogen) according to the manufacturer's protocol. 60 hours after transfection, cells were harvested for analysis.

### Cell proliferation assays

Cell proliferation in two dimensional culture or in Matrigel was analyzed as we described previously [19].

### BrdU incorporation assay

Two days after transfection, cells were serum-starved overnight and then incubated with BrdU (10 μM) in DMEM containing 10% FBS for 8 hr. The amount of BrdU incorporated into cells was determined by using a mouse anti-BrdU antibody, followed by an Alexa 488-conjugated goat anti-mouse IgG secondary antibody [35]. DAPI was used to stain nuclear DNA. At least five images per slide, at random fields, were taken by a Leica epifluorescence microscope using a X20 lens and analyzed by the ImageJ software.

### Cell migration and invasion assay

Transwell migration of MDA-MB-231, MCF7 and BT549 was determined using a 96 well-modified Boyden chamber [19]. For the invasion assay, a 24-well transwell insert (Corning Costar), coated with a thin-layer of growth factor-reduced Matrigel was used, and cell invasion was carried out at 37°C for 20 hr.

### Wound healing assay

A tumor cell wound-healing assay was performed in DMEM containing 10% FBS with 10μg/ml mitomycin as previously described [19].

### Measurement of cyclic AMP and cytosolic Ca^2+^ concentration

The level of cAMP and cytosolic concentration of Ca^2+^ ([Ca^2+^]_i_) in MDA-MB-231 cells was measured as previously described [19].

### Immunoprecipitation

MDA-MB231 cells were transduced with adenoviruses encoding FLAG-GFP or FLAG-WDR26. Two days after transduction, cells were serum-starved overnight, and then stimulated with LPA (10 μM) for the indicated time. Cells were lysed in modified RIPA buffer (without SDS), and the interaction between WDR26 and endogenous Gβγ, PI3K, and AKT was assessed by a co-immunoprecipitation as we reported [22, 24]. Similar immunoprecipitation assays were used to assess the interaction of HA-PI3Kβ with myc-WDR26 and FLAG-Gβ1γ2 for the formation of a signaling complex, and the interaction of FLAG-WDR26 deletion mutants with HA-PI3Kβ in MDA-MB231 cells transduced with adenoviruses encoding genes for these proteins. Similar assays were used to assess the interaction of HA-AKT2 with myc-WDR26 and FLAG-Gβ1γ2 for the formation of a signaling complex, and the interaction of HA-AKT2 with FLAG-WDR26 mutants, except that these proteins were transiently expressed in HEK293 cells using the Polyjet DNA *in vitro* transfection reagent (SignaGen).

### Xenograft mouse models

All animal studies were conducted in accordance with an IACUC-approved protocol (4091161) at the University of Iowa. Female nude BALB/c mice, aged 8 to 10 weeks, were used for the study. 1 ×10^6^ of MDA-MB-231 cells expressing inducible EGFP, WDR123-661 and WDR295-661 in 100μl saline were implanted into the right inguinal mammary fat pads of mice that were randomly chosen. They were allowed to grow for 23 days to form palpable primary tumors before the mice were treated with doxycycline (2mg/ml in drinking water) to induce protein expression. Primary tumor growth was monitored by bioluminescence imaging (BLI) and caliper measurement, weekly, by an investigator **blinded** to the experimental groups [19]. Once the primary tumor volume reached ~500 mm^3^, they were resected and the mice were monitored for another four weeks, for the formation of metastases by BLI. The metastases were confirmed by post-mortem *ex-vivo* BLI.

### Statistical analysis

Survival curves were analyzed according to the Kaplan-Meier method using the Graphpad Prism 6 Software, and the differences between curves were evaluated by the log-rank test. To compare xenograft tumor growth rate after doxycycline treatment, the tumor size, depicted as the bioluminescent photo counts, were log transformed, and plotted against time. A linear regression analysis was performed using the Graphpad Prism 6 Software, to obtain the slopes, and the difference in the slope values were evaluated by the analysis of covariance. Data are expressed as mean ± S.E.M. Means between two groups were compared with a two-tailed, unpaired Student's *t* test, while comparisons of means from multiple groups with each other or against one control group were analyzed with one-way ANOVA, followed by Tukey's range test. A p value of less than 0.05 was considered to be statistically significant.
